# Supplementary material for: Clinical and bioethical implications of health care interruption during the COVID-19 pandemic: A cross-sectional study in outpatients with rheumatic diseases
Source: PLoS One. 2021 Jul 9;16(7):e0253718. doi: 10.1371/journal.pone.0253718 (PMC8270122; doi:10.1371/journal.pone.0253718)
Supplement: S1 Table — (PDF) [file pone.0253718.s002.pdf]

Supplementary Table 1. N° (%) of patients with a face-to-face consultation at the OCDIR, with the ten most frequent rheumatic diagnoses specified.

**Specific rheumatic diagnoses[1]**	**Nº (%) of patients N=670**
Systemic Lupus Erythematosus	285 (42.5)
Rheumatoid arthritis	223 (33.3)
Systemic Vasculitis	32 (4.8)
Sclerodermia	22 (3.3)
Spondyloarthritis	21 (3.1)
Overlap syndromes	20 (3)
Inflammatory Myopathies	13 (1.9)
Primary Anti-Phospholipid Syndrome	9 (1.3)
Primary Sjögren Syndrome	8 (1.2)
IgG4-related disease	8 (1.2)
Other diagnosis	27 (4.0)

[1]*Diagnosed based on the attending rheumatologist criteria.*
