# Supplementary material for: Clinical and bioethical implications of health care interruption during the COVID-19 pandemic: A cross-sectional study in outpatients with rheumatic diseases
Source: PLoS One. 2021 Jul 9;16(7):e0253718. doi: 10.1371/journal.pone.0253718 (PMC8270122; doi:10.1371/journal.pone.0253718)
Supplement: S2 Table — (PDF) [file pone.0253718.s003.pdf]

Supplementary table 2. Regression analysis to identify factors associated with HCI when rheumatic disease clinical status was assessed by an independent observer according to pre-specified criteria (115 missing data [17%] due to non-access to charts review).

	**OR**	**95% CI**	**p**
Non-RA diagnosis	2.45	1.67-3.61	≤0.001
Rheumatic disease comorbidity index score ≥1	1.66	1.16-2.38	0.005
Patient´s need for rheumatic medical care	2.67	1.66-4.30	≤0.001
Adequate control of the rheumatic disease	0.58	0.38-0.87	0.010

OR=0dds Ratio; CI=confidence interval; RA=rheumatoid arthritis. R^2*=0.078*
